# 1060. Pritelivir in Immunocompromised Patients with Mucocutaneous Acyclovir-Resistant Herpes Simplex Virus-Infections – First Case Series

**DOI:** 10.1093/ofid/ofab466.1254

**Published:** 2021-12-04

**Authors:** Kim WorkowskiJoerg Albrecht, Robin K Avery, Robin K Avery, Pranatharthi Chandrasekar, Roy F Chemaly, Nicolas C Issa, Camille Kotton, Camille Kotton, Princy N Kumar, Ramesh Mayur, Moti Ramgopal, Joshua Schiffer, Anna Wald, Michael G Ison

**Affiliations:** 1 Cook County Health, Chicago, Illinois; 2 Johns Hopkins, Baltimore, MD; 3 wayne state university, detroit, MI; 4 The University of Texas MD Anderson Cancer Center, Houston, TX; 5 Brigham and Women’s Hospital, Boston, Massachusetts; 6 Massachusetts General Hospital, Boston, MA; 7 Georgetown University School of Medicine, Washington, District of Columbia; 8 Henry Ford Hospital, Detroit, MI; 9 Midway Research Center, Ft. Pierce, FL; 10 Fred Hutch, Seattle, Washington; 11 University of Washington, Seattle, Washington; 12 Northwestern University, Chicago, IL

## Abstract

**Background:**

HSV recurrences are usually managed effectively with existing antiviral drugs (nucleoside analogs such as acyclovir). However, in immunocompromised patients (e.g., malignancy, HIV, transplant), if lesions persist or recur while receiving antiviral treatment, acyclovir resistance should be suspected. In this population, there are limited treatment options. The helicase-primase inhibitor pritelivir is a novel oral antiviral, with a new mode of action and is active against both HSV-1 and HSV-2, including acyclovir and foscarnet-resistant strains. In this case series, we report the first clinical experiences with pritelivir in the treatment of immunocompromised patients with acyclovir resistant HSV infection.

**Methods:**

All patient reported in this case series received pritelivir in a Phase 2 study. There were treated in an open-label design with a 400 mg pritelivir oral loading dose followed by a 100 mg oral maintenance dose daily for up to 28 days.

**Results:**

Of the 23 patients, 11 had HIV infection and 12 had malignancy, transplant or an autoimmune disease. Of this cohort, 19 patients showed full resolution of their HSV-related lesions during the 28 day treatment period, while in 4 subjects lesions improved but did not completely heal during the observation period. Pritelivir was well tolerated without significant adverse effects.Reasons for incomplete lesion resolution during the 28 day treatment period, were extensive lesions in one patient, one patient with resistance development, and one patient with lesions in the oral cavity. Three patients subsequently experienced full resolution, while one patient required foscarnet due to CMV reactivation, necessitating early discontinuation.

**Conclusion:**

Pritelivir is a promising novel treatment option for patients with severe mucocutaneous HSV-1/2 infections that are resistant to acyclovir and foscarnet. An international Phase 3 study is underway to evaluate pritelivir efficacy in immunocompromised patients.

**Disclosures:**

**Joerg Albrecht, MD/PhD**, **Biogen** (Scientific Research Study Investigator)**Investigator for AiCuris** (Scientific Research Study Investigator) **Robin K. Avery, MD**, **Aicuris** (Grant/Research Support)**Astellas** (Grant/Research Support)**Chimerix** (Research Grant or Support)**Merck** (Grant/Research Support)**Oxford Immunotec** (Grant/Research Support)**Qiagen** (Grant/Research Support)**Takeda/Shire** (Grant/Research Support) **Roy F. Chemaly, MD, MPH, FACP, FIDSA**, **AiCuris** (Grant/Research Support)**Ansun Biopharma** (Consultant, Grant/Research Support)**Chimerix** (Consultant, Grant/Research Support)**Clinigen** (Consultant)**Genentech** (Consultant, Grant/Research Support)**Janssen** (Consultant, Grant/Research Support)**Karius** (Grant/Research Support)**Merck** (Consultant, Grant/Research Support)**Molecular Partners** (Consultant, Advisor or Review Panel member)**Novartis** (Grant/Research Support)**Oxford Immunotec** (Consultant, Grant/Research Support)**Partner Therapeutics** (Consultant)**Pulmotec** (Consultant, Grant/Research Support)**Shire/Takeda** (Consultant, Grant/Research Support)**Viracor** (Grant/Research Support)**Xenex** (Grant/Research Support) **Nicolas C. Issa, MD**, **AiCuris** (Scientific Research Study Investigator)**Astellas** (Scientific Research Study Investigator)**GSK** (Scientific Research Study Investigator)**Merck** (Scientific Research Study Investigator) **Camille Kotton, MD**, **Shire/Takeda** (Advisor or Review Panel member) **Camille Kotton, MD**, UpToDate (Individual(s) Involved: Self): I write chapters on zoonoses for UpToDate., Independent Contractor **Princy N. Kumar, MD**, **AMGEN** (Other Financial or Material Support, Honoraria)**Eli Lilly** (Grant/Research Support)**Gilead** (Grant/Research Support, Shareholder, Other Financial or Material Support, Honoraria)**GSK** (Grant/Research Support, Shareholder, Other Financial or Material Support, Honoraria)**Merck & Co., Inc.** (Grant/Research Support, Shareholder, Other Financial or Material Support, Honoraria) **Moti Ramgopal, MD FACP FIDSA**, **Abbvie** (Scientific Research Study Investigator, Speaker’s Bureau)**Gilead** (Consultant, Scientific Research Study Investigator, Speaker’s Bureau)**Janssen** (Consultant, Scientific Research Study Investigator, Research Grant or Support, Speaker’s Bureau)**Merck** (Consultant, Scientific Research Study Investigator)**ViiV** (Consultant, Scientific Research Study Investigator, Speaker’s Bureau) **Anna Wald, MD, MPH**, **Aicuris** (Consultant)**Crozet** (Consultant)**GSK** (Scientific Research Study Investigator)**Merck** (Other Financial or Material Support, DSMB)**Sanofi** (Scientific Research Study Investigator)**X-Vax** (Consultant) **Michael G. Ison, MD, MS**, **Celltrion, Inc.** (Consultant)

